# Histopathological and Molecular Features of Adenomatous and Serrated Colon Adenomas, Characteristics and Overlapping Features, Challenges in the Nomenclature

**DOI:** 10.5146/tjpath.2026.13786

**Published:** 2026-09-15

**Authors:** Buket Bambul Sıgırcı, Ismail Yılmaz, Sibel Erdamar Çetin, Enver Yarıkkaya, Merve Cin, Esra Pasaoglu, Nevra Dursun

**Affiliations:** Department of Pathology, Sisli Hamidiye Etfal Training and Research Hospital, İstanbul, Türkİye; Department of Pathology, Sultan Abdulhamid Han Training and Research Hospital, İstanbul, Türkİye; Department of Pathology, Acıbadem University,İstanbul, Türkİye; Department of Pathology, Istanbul Training and Research Hospital, İstanbul, Türkİye; Department of Pathology, Istanbul Training and Research Hospital, İstanbul; Türkİye,Department of Pathology, Bağcılar Training and Research Hospital, İstanbul, Türkİye; Department of Pathology, Basaksehir Cam ve Sakura City Hospital, İstanbul, Türkİye

**Keywords:** Colorectal adenomas, Sessile serrated lesion, Traditional serrated adenoma, Ectopic crypt, Microsatellite Instability

## Abstract

**Objective:** Colorectal cancer develops from precursor epithelial polyps, including tubular adenomas, villous or tubulovillous adenomas, sessile serrated lesions, and traditional serrated adenomas. Although diagnostic criteria for these lesions have been established, overlapping features often complicate classification. This study aimed to define the characteristics and shared features of colorectal adenomas.

**Material and Methods:** A total of 140 adenomas were evaluated, comprising 71 conventional adenomas, 34 sessile serrated lesions, and 35 traditional serrated adenomas. Hematoxylin and eosin–stained slides were reviewed. Macroscopic features, including sessile or polypoid structure, were documented from pathology and endoscopy reports. Histopathological features were assessed first as present or absent, and then by extent using a four-tiered scale (0: less than 10%, 1: 10–25%, 2: 25–75%, 3: more than 75%). Sixteen adenomas with overlapping features were classified as hybrid or unclassified. Molecular studies, including mutation analysis of KRAS, NRAS, and BRAF, as well as microsatellite instability and MLH1 promoter methylation, were performed in 50 cases.

**Results:** No gender predominance was identified. Sessile morphology was most common in sessile serrated lesions and hybrid adenomas. Conventional adenomas showed serration in 50% of cases and ectopic crypts in 23%, though usually involving less than 25% of the lesion. Adenomatous dysplasia was present in most traditional serrated adenomas and nearly half of sessile serrated lesions, while serrated dysplasia occurred in a minority of conventional adenomas. KRAS mutations predominated in conventional (55%) and hybrid adenomas (80%), whereas BRAF mutations were most frequent in sessile serrated lesions (60%) and traditional serrated adenomas (40%); MLH1 promoter methylation was observed across all types, while no NRAS mutations or microsatellite instability were detected.

**Conclusion:** Histopathological features overlapped among all adenoma types, and no single feature was lesion-specific. Applying quantitative thresholds may improve diagnostic accuracy and reduce interobserver variability.

## INTRODUCTION

Adenomas are the most common neoplasms in the colon and are frequently implicated in the development of colon cancer (1,2). Two classical morphological pathways (classical adenoma-carcinoma development and serrated neoplasia development) and three molecular pathways (chromosomal instability, microsatellite instability, and CpG island methylation) have been identified as playing a role in the progression from adenoma to carcinoma (3,4). The latest updates of the WHO 2019 Gastrointestinal System Tumors manual have updated the precursors of these pathways for conventional adenomas (tubular, villous and tubulovillous adenoma) and serrated lesions (hyperplastic polyp, sessile serrated lesion, dysplastic sessile serrated lesion, traditional serrated adenoma, unclassified serrated adenoma) (2). In this most recent version, the histological characteristics of adenomas were also determined. Nevertheless, the book and the literature are unclear about the differential diagnosis of these adenomas when there are overlapping histological features (5). The focus of many recent studies has been on distinguishing between hyperplastic polyps and neoplastic serrated lesions. Additionally, the diagnosis of lesions displaying mixed characteristics, such as conventional adenoma with serrated features, poses challenges for pathologists in their routine practice (6,7). Studies in the literature have reported that adenomatous dysplasia can be seen in serrated neoplasms at varying rates and serrated dysplasia can also be seen in conventional adenomas. However, there is no information on how to make the differential diagnosis of these lesions when dysplasia is seen (8). Furthermore, there is a lack of clarity regarding the specific histopathological features that should be considered when naming a lesion and the extent to which these features contribute to meeting diagnostic criteria. Therefore, the aim of this study was to investigate whether the histopathological and molecular features of polyps/adenomas from either pathway can aid in the differentiation and diagnosis of these lesions.

## MATERIAL and METHODS

### Case Inclusion and Exclusion Criteria

A total of 140 cases were included in this retrospective study, comprising 71 conventional adenomas (CA) (35 tubular adenomas (TA) and 36 tubulovillous adenomas (TVA)), 34 sessile serrated lesions (SSL), and 35 traditional serrated adenomas (TSA), as diagnosed at our clinic. Each case underwent re-evaluation by one experienced pathologist, two moderately experienced pathologists, and one junior pathologist. To be included in the study, each case had to have sufficient tissue in the blocks for molecular tests. Additionally, for microsatellite instability (MSI) analysis, it was required that at least 10 cases had normal tissue, or a biopsy with normal tissue had been obtained from another region at a different time. Cases where access to the archival block was not possible or those that had been sent to an external center for consultation were excluded.

### Demographic and Histopathological Analysis

Data regarding age, gender, lesion size, localization, and pedunculated or sessile status were obtained from the hospital intranet system. All pathology materials from the cases were re-evaluated, and the following features were assessed: tubular structure, villous structure, serration (slit-like and usual), ectopic crypt, eosinophilic cytoplasm, pencillate nucleus, crypt base dilatation, mucinous hypersecretion at the base and surface, adenomatous/serrated dysplasia, and the degree of dysplasia. The presence of tubular and villous structures, serration, ectopic crypt, eosinophilic cytoplasm, pencillate nucleus, and mucinous hypersecretion at the base and surface were initially evaluated using a two-item system (present/absent), and then a four-item scoring system was employed to determine the extent of these features relative to the total material area (0: <10%, 1: 10-25%, 2: 25-75%, 3: 75% or more). Adenomatous and serrated dysplasia, as well as dilatation of the crypt base, were assessed as present/absent, as one basal dilatation in a crypt was sufficient for serrated lesions according to the 2019 WHO classification.

A diagnosis of traditional serrated adenoma was based on the presence of luminal slit-like serration, ectopic crypt, eosinophilic cytoplasm, pencillate nucleus, and for serrated lesions serration with mucin droplets and goblet cells in the crypts, crypt base dilatation (observed as an inverse T or L shape), asymmetrical proliferation, and horizontal growth along the muscularis mucosa were used (2,9,10). A diagnosis of conventional adenoma was established by identifying spindling of the epithelium with loss of polarity, varying degrees of hyperchromatic features, nuclei with increased stratification, and a reduced number of goblet cells and absorptive cells. Subtyping of conventional adenomas was performed based on the degree of tubular and villous architecture (2). The final diagnosis was determined when at least three pathologists reached the same conclusion. Cases where a majority consensus could not be reached were classified as hybrid/unclassified cases.

### Molecular Analysis

Molecular analysis, including KRAS, NRAS, BRAF mutation analysis, microsatellite instability analysis, and MLH-1 promoter methylation analysis, was conducted on a total of 50 cases, with 10 cases from each group (TA, TVA, SSL, TSA, and hybrid) having sufficient tissue in the paraffin blocks.

BRAF, KRAS, NRAS Mutation Analyses: DNA Extraction: Ten histological tissue sections with a thickness of 10 µm were obtained from the extra-lesional formalin-fixed paraffin-embedded tissue blocks of each patient for lesion and microsatellite instability analysis. The sections were manually located to include the areas that best represented the lesion. The dissected tissue sections were placed in a sterile 1.5 ml Eppendorf tube, and the paraffin was removed. DNA extraction was performed using the QIAamp DNA FFPE Tissue Kit (Catalog No: 56404). The quantity and quality of the extracted DNA were measured using a spectrophotometer, and the DNA was stored at -20°C until further analysis.

PCR and Sequencing: Exon 15 of the BRAF gene, exon 2, 3, and 4 of the NRAS gene, and exon 2, 3, and 4 of the KRAS gene were amplified using PCR with the HotStarTaq DNA Polymerase kit (Qiagen, Germany, Catalog No: 203205) and the specified forward and reverse primers listed in (Table 1). Once it was confirmed that the samples had successfully amplified to the expected length, the control was functional, and there was no contamination, the PCR products underwent purification according to the protocol using the QIAquick PCR Purification Kit (Qiagen, Germany, Catalog No: 28106). The purified PCR products were then subjected to forward and reverse sequencing following the protocol with the Big Dye Terminator v 3.1 Cycle Sequencing kit using the ABI-3730 (48 capillaries) DNA Sequencer device.

**Table 1:** Forward and reverse primer sequences of exon

**Primer sequences of each exon.**		
**Gene**	**Forward primer sequence (5’→3’)**	**Reverse primer sequence (5’→3’)**
BRAF exon 15	TCATAATGCTTGCTCTGATAGG	GGCCAAAAATTTAATCAGTGG
KRAS exon 2	GTGTGACATGTTCTAATATAGTCA	CTGTATCAAAGAATGGTCCTGCAC
KRAS exon 3	TTTTTGAAGTAAAAGGTGCACTG	TTTAAACCCACCTATAATGGTGAA
KRAS exon 4	GGACTCTGAAGATGTACCTATGGTC	AAGAAGCAATGCCCTCTCAA
NRAS exon 2	GAAAGCTTTAAAGTACTGTAGATGTGG	AGATGATCCGACAAGTGAGAGA
NRAS exon 3	CCCCTTACCCTCCACACC	CACAAAGATCATCCTTTCAGAGAA
NRAS exon 4	TGGTGCTAGTGGGAAACAAG	TGAATATGGATCACATCTCTACCA

Microsatellite Instability and MLH-1 Promoter Methylation Analysis: DNA extraction from tissues with or without tumor cells was completed in the previous step. The PCR master mix, positive and negative controls, sample without DNA, and K 562 (used as an amplification control) were distributed among the patient samples for the amplification procedure. PCR amplification was performed using the Promega MSI Analysis System, Version 1.2 kit, with the 5 mononucleotide repeat markers (NR-21, BAT-25, MONO-27, BAT-26, and NR-24) for detecting microsatellite instability (MSI) and the 2 pentanucleotide repeat markers (Penta C and Penta D) for assessing consistency between the patient’s lesion and normal tissue and detecting any potential contamination. The fluorescence-labeled PCR products were then analyzed and sized using electrophoresis in the ABI-3730 (48 capillaries) automatic sequencing device. The genotypes for each marker were compared and scored as either stable or unstable. MLH-1 promoter analysis was performed using the Real-Time PCR method. Methylation of the MGMT gene was detected in the promoter region and at the 9 CpG islands in exon-1.

### Statistical Method

Continuous variables were presented as mean and standard deviation (SD), while categorical variables were presented as number and percentage. The normality of numerical variables was assessed using the Kolmogorov-Smirnov test. For normally distributed data, the independent samples t-test was used for analysis, while the Mann-Whitney U test was used for data with skewed distribution. Categorical variables were presented as percentages and analyzed using the chi-square test. One-way analysis of variance (ANOVA) was used to assess statistically significant differences among more than two independent (unrelated) groups for normally distributed data, while the Kruskal-Wallis test was used for data with an abnormal distribution. The Tukey test was employed to compare differences between parametric variables, and the Dwass-Steel-Critchlow-Fligner test was used for non-parametric variables. Pearson’s chi-square test was used to compare differences between categorical variables. Pearson correlation analysis was used for variables with normal distribution, while Spearman correlation analysis was used for variables with abnormal distribution to evaluate the relationships between variables. All statistical analyses were performed using Jamovi (Version 0.9), a computer software for statistical analysis (Jamovi project, 2018). A p-value < 0.05 was considered statistically significant.

## RESULTS

Microscopic evaluation led to a change in the initial diagnosis for 10 cases initially diagnosed as TVA, 5 cases as SSL, and 1 case as TSA, resulting in a reclassification as hybrid adenomas (HA). Consequently, the number of cases for CA, SSL, TSA, and HA was determined as 61, 29, 34, and 16, respectively.

(Table 2) shows the demographic, histopathological and molecular findings of each group.

**Table 2:** Demographic, histopathological and molecular findings of all groups

**Findings**	**CA n=61**	**SSL n=29**	**TSA n=34**	**HA n=16**	**p-value**
	**n (%)**	**n (%)**	**n (%)**	**n (%)**	
Sex (male)	45/61 (73.7)	18/29 (62)	23/34 (67.6)	11/16 (68.7)	0.7222
Age (year)	63 (55-69)	59 (54-71)	65 (59-74)	64 (56-70)	0.2221
Lesion diameter (cm)	1.20 (1-1.73)	0.8 (0.5-1.5)	1.5 (1-2.5)	1.45 (0.94-2.12)	0.0011
Sessile morphology	12/61 (19.6)	29/29 (100)	18/34 (52.9)	12/16 (75)	<0.0012
Left-sided localization	53/61 (86.8)	12/29 (41.3)	28/34 (82.3)	14/16 (87.5)	<0.0012
Tubular structures	61/61 (100)	21/29 (72.4)	19/34 (55.8)	16/16 (100)	<0.0012
Villous morphology	27/61 (44.2)	18/29 (62)	27/34 (79.4)	10/16 (62.5)	0.0092
Basal mucinous hyperplasia	16/61 (26.2)	13/29 (44.8)	2/34 (5.8)	2/16 (12.5)	0.0022
Luminal mucinous hyperplasia	6/61 (9.8)	22/29 (75.8)	1/34 (2.9)	2/16 (12.5)	<0.0012
Serration	1/61 (1.6)	29/29 (100)	32/34 (94.1)	9/16 (56.2)	<0.0012
Ectopic crypts	2/61 (3.2)	0/29 (0)	18/34 (52.9)	2/16 (12.5)	<0.0012
Eosinophilic cytoplasm	2/61 (3.2)	2/29 (6.8)	16/34 (47)	2/16 (12.5)	<0.0012
Slender nuclei	4/61 (6.5)	1/29 (3.4)	13/34 (38.2)	2/16 (12.5)	<0.0012
Adenomatous dysplasia (AD)	61/61 (100)	16/29 (55.1)	23/34 (67.6)	16/16 (100)	<0.0012
Serrated dysplasia (SD)	6/61 (9.8)	5/29 (17.2)	14/34 (41.1)	4/16 (25)	0.0042
Low grade AD	58/61 (95)	14/29 (48.2)	22/34 (64.7)	16/16 (100)	<0.0012
High grade AD	33/61 (54)	5/29 (17.2)	6/34 (17.6)	8/16 (50)	<0.0012
Low grade SD	6/61 (9.8)	5/29 (17.2)	14/34 (41.1)	2/16 (12.5)	0.0022
High grade SD	2/61 (3.2)	1/29 (3.4)	5/34 (14.7)	4/16 (25)	0.0162
Basal dilatation	16/61 (26.2)	28/29 (96.5)	18/34 (52.9)	12/16 (75)	<0.0012
Filiform morphology	6/61 (9.8)	0/29 (0)	8/34 (23.5)	3/16 (18.7)	0.0282
BRAF mutation	0/20 (0)	6/10 (60)	4/10 (40)	1/10 (10)	0.0012
KRAS mutation	11/20 (55)	2/10 (20)	6/10 (60)	8/10 (80)	0.0572
NRAS mutation	0/20 (0)	0/10 (0)	0/10 (0)	0/10 (0)	0.1122
Microsatellite instability (MSI)	0/20 (0)	0/10 (0)	0/10 (0)	0/10 (0)	0.1122
Methylation	4/20 (20)	4/10 (40)	2/10 (20)	2/10 (20)	0.6252
*1Kruskal-Wallis; 2Pearson*					

### Demographic Findings

Out of the total 140 patients, 43 (30.7%) were females and 97 (69.3%) were males, with an age range of 27-88 years and a mean (SD) age of 63.3 (±11.22) years. There were no statistically significant differences (p > 0.05) in terms of age and gender among the CA, SSL, TSA, and hybrid groups.

### Histopathological Findings

SSLs exhibited a higher rate of right colon localization compared to other groups (p < 0.05). Furthermore, both SSLs and the hybrid group showed a higher prevalence of sessile macroscopic configuration (p < 0.05).

**Figure 1:**
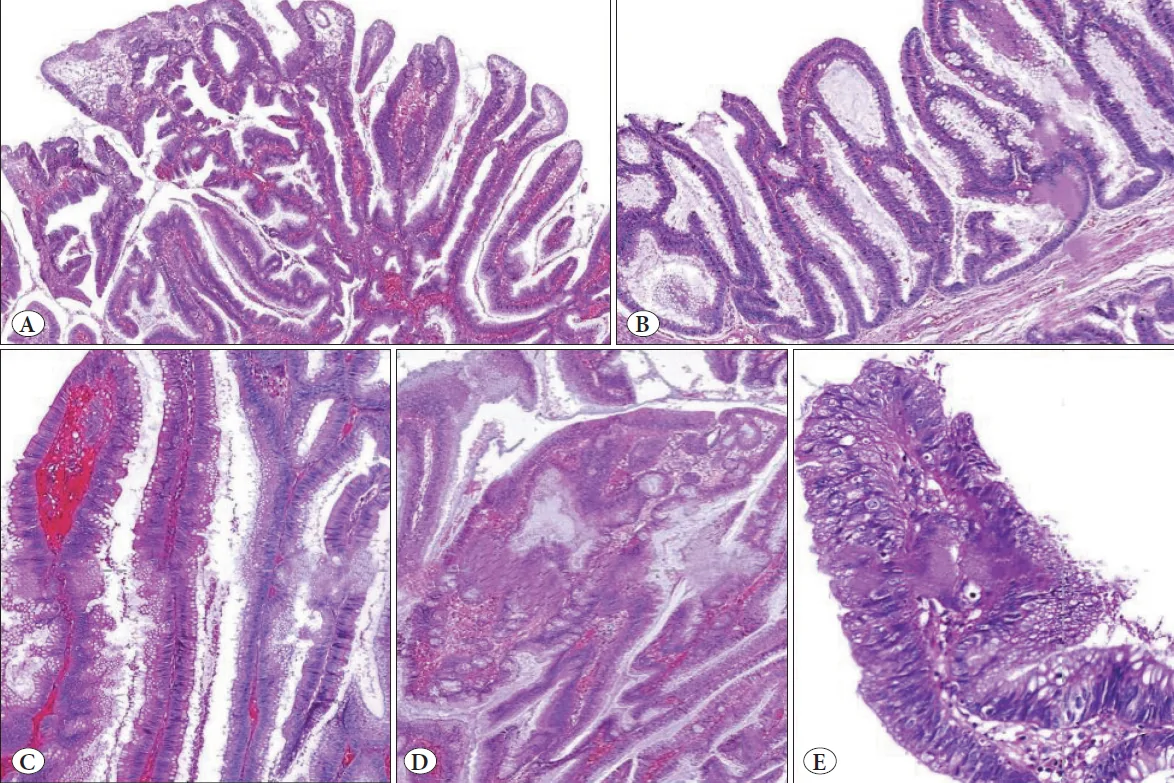
A) Focal serration, B) Dilatation at the crypt base C) Eosinophilic cytoplasm and pencillate nucleus, D) Ectopic crypts, E) Focal serrated dysplasia in conventional adenoma (A:10X, B:10X, C:20X, D:20X, E: 40X; H&E).

Adenomatous dysplasia was the characteristic of conventional adenomas (p < 0.001), with serrated dysplasia observed in 10% of the cases. Luminal serration was present in more than half of the CA cases, but its occurrence was 25% or higher in only 3 cases. Eosinophilic cytoplasm (n=30), pencillate nucleus (n=20), and ectopic crypt (n=23) were frequently observed in CA cases, but with a score of 1 or 0 in the majority. Crypt base dilatation was identified in 16 of the CA cases. (Figure 1).

**Figure 2:**
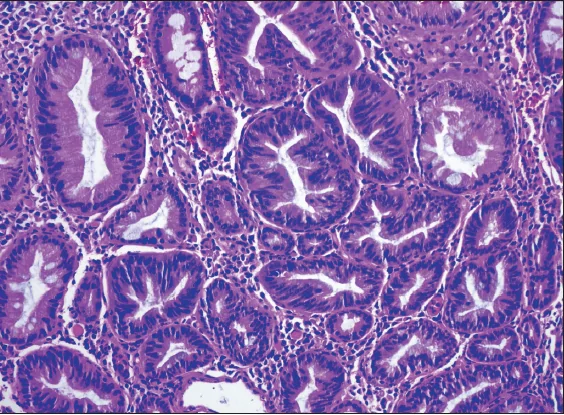
Figure 2: Sessile serrated lesion with adenomatous dysplasia (40X, H&E).

For SSLs, the most distinguishing features were serration, dilated crypt base, and mucinous hypersecretion (p < 0.001). At least focal adenomatous dysplasia (low- or high-grade) was present in over half (55%) of SSL cases, dysplasia was observed in only five cases (17%) (Figure 2). Ectopic crypt (n=4, 14%) and pencillate nucleus (n=5, 17%) were rare findings and localized in focal areas within SSLs.

**Figure 3:**
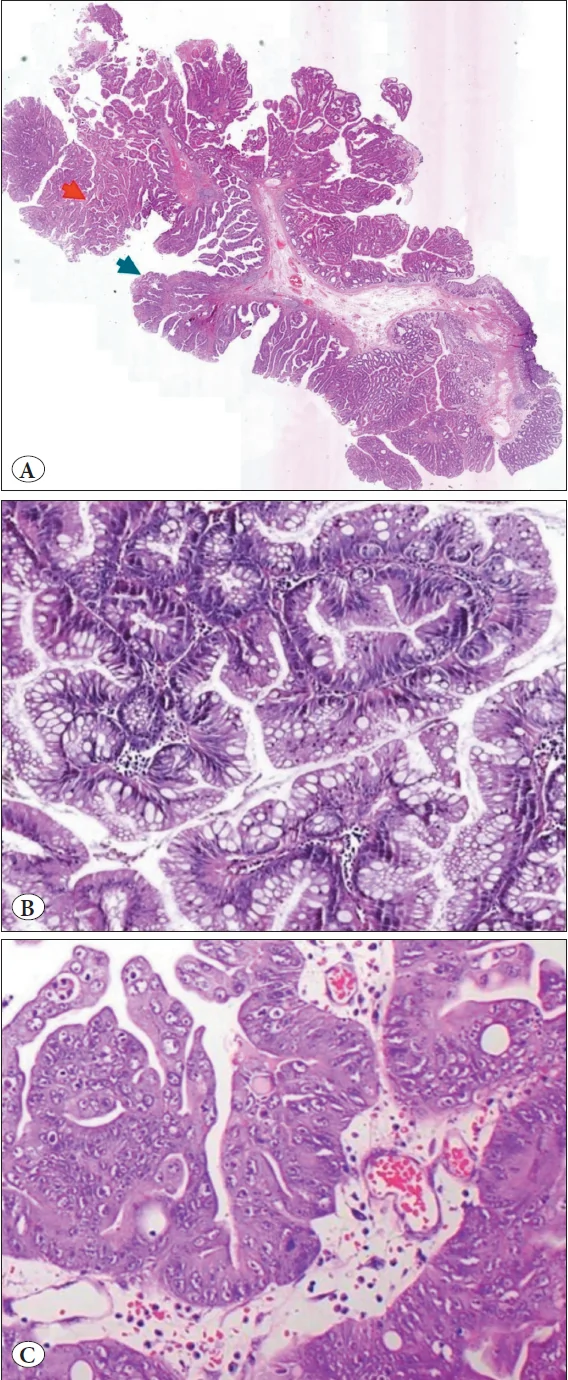
Traditional serrated adenoma with dysplasia (red arrow shows area with adenomatous dysplasia; blue arrow shows area with serrated dysplasia) (A) with adenomatous dysplasia (B) and with serrated dysplasia, the area shown with arrow (C) (A: 10X, B,C: 40X, H&E).

TSAs were characterized by luminal slit-like serration, ectopic crypt, pencillate nucleus and eosinophilic cytoplasm (p < 0.001). Adenomatous dysplasia was found in 67% of TSA cases, serrated dysplasia in 41%, and crypt base dilatation in 52% (Figure 3).

Among the 16 polyps classified as hybrid adenomas due to the absence of a definitive diagnosis, 11 were sessile. All cases in this group exhibited adenomatous dysplasia, and 25% had concurrent serrated dysplasia. Luminal serration was observed in all cases, while crypt base dilatation was present in 75%.

Among the histopathological criteria, the presence of luminal serration in CAs was below 25%. By establishing a threshold value of 25% for the presence of luminal serration (≥25% as present, <25% as absent), we found it to be a useful criterion for distinguishing CAs from serrated lesions (p < 0.001) (Graph I).

**Graph 1:**
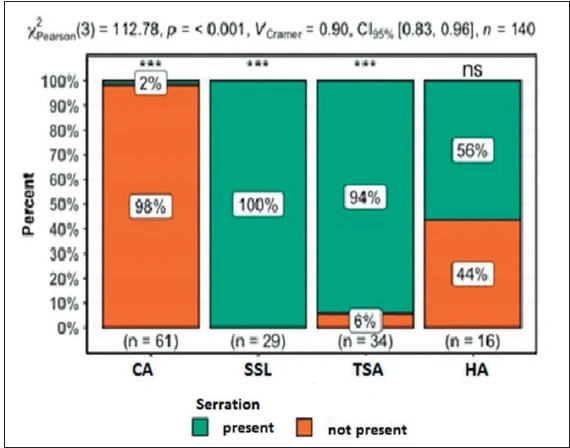
The chart shows that after using a cut off of %25 for serration, CAs can be differentiated from the serrated ones.

Similarly, when using a threshold value of 10% for the presence of pencillate nucleus and ectopic crypts in serrated lesions (≥10% as present, <10% as absent), we were able to differentiate TSAs from SSLs. TSAs comprised over 95% of the polyps with a luminal serration rate > 25%, ectopic crypt rate > 10%, and pencillate nucleus rate > 10% (p < 0.001) (Graph II).

**Graph 2:**
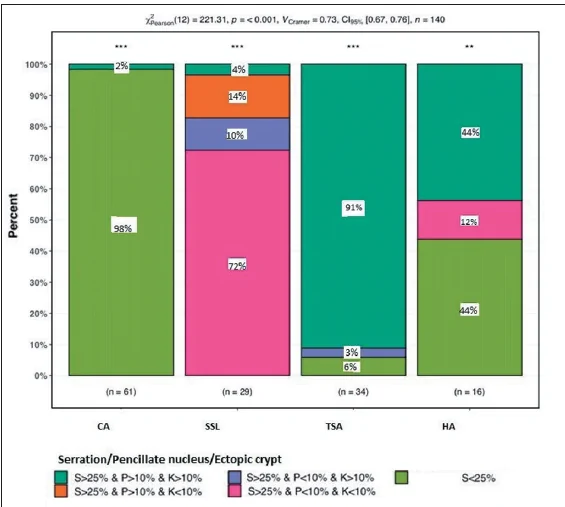
The chart shows that after using a cut off of 25% for serration and 10% for pencillate nucleus and ectopic crypt, TSAs can be differentiated from the others.

**Figure 4:**
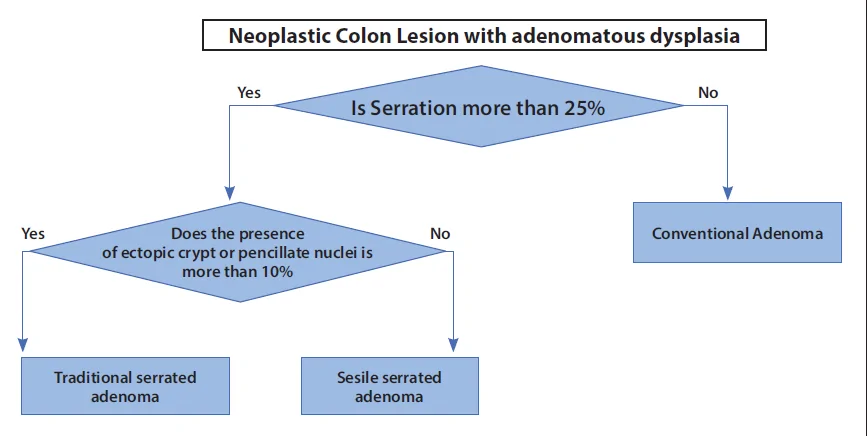
Neoplastic colon polyp with adenomatous dysplasia

Based on these threshold values, we developed an algorithm for cases with adenomatous dysplasia that posed a diagnostic challenge (Figure 4). By using this algorithm, 7 cases originally classified as unclassified could be reclassified as CA, all of which were found to have KRAS mutations. Two cases originally classified as unclassified could be reclassified as SSA, with only one case having undergone molecular analysis, revealing a BRAF mutation. Seven hybrid cases initially classified as TSA according to this algorithm had 3 cases with KRAS mutation. Additionally, 8 cases originally classified as SSA were reclassified as TSA, with one case showing focal TSA features upon reevaluation. Only 2 cases had undergone molecular analysis, both of which had KRAS mutations, and no further material was available for examination after molecular analysis in the other block.

### Molecular Findings

Among the cases diagnosed with CA, SSL, TSA, and hybrid adenoma, no NRAS mutations or microsatellite instability were observed in 20, 10, 10, and 10 cases, respectively. BRAF mutations were found in 6 SSLs, 4 TSAs, and 1 hybrid adenoma, while KRAS mutations were found in 11 CAs, 2 SSLs, 6 TSAs, and 8 hybrid cases. MLH1 promoter methylation was detected in 4 CAs, 4 SSLs, 2 TSAs, and 2 hybrid cases. (Table 3).

**Table 3:** Mutation analyses

	**BRAF**	**KRAS**	**NRAS**	**MSI**	**MLH1-Methylation**
CA (n=20)	0	11	0	0	4
SSL (n=10)	6	2	0	0	4
TSA (n= 10)	4	6	0	0	2
HA(n=10)	1	8	0	0	2

**CA: **Conventional Adenomas, **SSL:** Sessile Serrated Lesions, **TSA: **Traditional Serrated Adenomas, **HA:** Hybrid Adenomas

Two cases diagnosed with SSL that exhibited the KRAS mutation were also found to have concurrent MLH-1 methylation. Histopathological examination of one of these cases revealed focal areas with histopathological characteristics resembling TSA. Most cases with MLH1 methylation demonstrated dysplastic changes.

## DISCUSSION

Most colorectal carcinomas arise from pre-cancerous polyps that are broadly categorized as either conventional adenomas or serrated polyps. The initial pathway elucidated in colorectal carcinogenesis was the “classical adenoma-carcinoma pathway,” characterized by microsatellite instability, chromosomal instability, and mutations in genes such as KRAS, APC, and TP53 (11). Subsequently, the “serrated neoplasia pathway” was identified as an alternative pathway, characterized by CpG gene promoter hypermethylation, along with mutations in TP53, KRAS, and BRAF, as well as microsatellite instability. As is well-known, three types of precancerous mass-producing lesion have been identified: conventional colorectal adenoma, sessile serrated adenoma, and traditional serrated lesion/polyp, which arise from these molecular pathways. Therefore, it is critical to correctly differentiate these types (12–15). The specific diagnostic histopathological characteristics of colon adenomas and dysplastic changes in these lesions have been documented (2,8). However, sometimes distinguishing between adenomas based on morphological characteristics can be challenging. Various lesions may exhibit similar histopathological changes, leading to difficulties in the differential diagnosis. Currently, there are no defined cut-off criteria for distinguishing such lesions, and pathologists require clear criteria for accurate definition and naming of adenomas. In addition, in recent years, it became more challenging to address lesions with serrated features. Numerous publications discuss new lesion definitions and kinds, as well as nomenclature (16–19). A study conducted by Ensari et al., focusing on 70 SSLs, examined whether European pathologists have reached a consensus regarding common diagnostic and nomenclature criteria. The study found that consensus among pathologists significantly increased when specific diagnostic criteria were provided in advance (20). Another study reported that TSAs with adenomatous dysplasia histopathologically resemble TVAs (referred to as serrated TVAs) and pose diagnostic challenges (21,22).

In our current study involving 140 adenomas, we assessed the histopathological parameters of all diagnostic groups to identify overlapping histopathological and molecular characteristics and establish clear criteria for diagnosing colorectal adenomas. We observed that all criteria, including those considered diagnostic, were present in varying proportions across all adenoma groups. None of the histopathological parameters were specific to a single type of lesion.

The key distinction between serrated lesions with dysplasia and conventional adenomas is the presence of serration. In our study, we observed at least focal serration in more than half of the conventional adenomas, but only a minority displayed serration involving more than 25% of the lesion. By analogy to the 25% cutoff used to distinguish tubulovillous from tubular adenomas, we propose that applying the same threshold would be a practical and broadly applicable approach for distinguishing serrated lesions with adenomatous dysplasia.

TSAs are complex and the most difficult lesions to differentiate, as in the literature (9,18,23–25). It is worth noting that the parameters considered characteristic features of TSAs, such as ectopic crypts, pencillate nucleus, and luminal serration, were found in all adenoma groups. However, like their molecular features, the histopathological distinctions of these lesions pose challenges.

Ectopic crypts, which are abnormal crypts that do not reach the muscularis mucosae and are considered definitive criteria for TSAs, were present in 23 cases of conventional adenomas, 4 cases of SSAs, and 11 cases in the hybrid/unclassified group. Several studies have compared the histopathological characteristics of TSAs with other adenomas and reported finding features of conventional adenomas and serrated lesions in TSAs and vice versa (22–24). Bakthiari et al. and Vayrynen et al. conducted separate studies and reported that ectopic crypts are predominantly associated with a villous morphology and can also be observed in conventional adenomas (25,26). In our current study, when we examined these three parameters, we found that luminal serration was present in most conventional adenomas but at a rate of 25% or less. Eosinophilic cytoplasm and pencillate nucleus were present in all groups but rarely exceeded 10% in conventional adenomas.

Bettington et al. have reported that for a diagnosis of TSA, at least two of the following three criteria should be present, with at least one criterion >50%: 1) typical cytology, 2) slit-like serration, and 3) ectopic crypts (27). Hiromoto et al. have diagnosed TSAs when all three of these criteria are present at 50% or more (28).

Similarly, in this study, we concluded that all three histopathological parameters needed to be present for a diagnosis of TSA. To differentiate TSAs from other adenomas, we established an algorithm using a threshold value of 25% for luminal serration (present > 25% and absent < 25%), and a threshold value of 10% for pencillate nucleus and ectopic crypts (present > 10% and absent < 10%) (Figure 4). This algorithm demonstrated that more than 95% of the polyps with luminal serration > 25%, ectopic crypts > 10%, and pencillate nucleus > 10% were TSAs.

In the studies conducted solely by Bettington and Hiromato, percentage values were used for the differentiation of lesions. In our study, we also developed a diagnostic algorithm using similar percentage values. However, while their study considered a threshold of 50% and above as significant, we determined 25% for serration and 10% for the presence of a pencillate core and ectopic crypts to be sufficient. Based on the results of our study, we suggest that this algorithm will aid in the diagnosis and classification of lesions.

When we applied this algorithm to our hybrid polyp group, the diagnosis was changed as 7 CAs, 7 TSAs, and 2 SSLs. Molecular evaluation performed on a subset of this group (n:10) revealed the presence of KRAS mutation in 6 cases of the CA subgroup, BRAF mutation in 1 case of the SSL subgroup (mutation analysis conducted in only 1 case), and KRAS mutation in 2 cases of the TSA subgroup (mutation analysis conducted in only 2 cases). BRAF mutations are most frequently observed in SSLs (60%), reinforcing their connection to the serrated pathway, while they are also found in 40% of TSAs, suggesting that some TSAs may originate from this pathway. In contrast, hybrid adenomas show a lower BRAF mutation rate (10%), indicating a mixed molecular background. KRAS mutations are predominantly present in CAs (55%) and HAs (80%), aligning them with the conventional adenoma-carcinoma sequence, while their presence in 60% of TSAs highlights their molecular heterogeneity and potential non-serrated origin. MLH1 promoter methylation, typically linked to the serrated pathway and often co-occurring with BRAF mutations, is found in 40% of SSLs and 20% of TSAs, suggesting its role in epigenetic regulation. Some SSL cases exhibiting both MLH1 methylation and KRAS mutations imply an alternative molecular mechanism, with TSA-like histological features supporting the hypothesis of SSL transformation into TSA. Hybrid adenomas, with a high KRAS mutation rate (80%) and a lower BRAF mutation rate (10%), appear more closely related to conventional adenomas, although their 20% MLH1 methylation suggests some overlap with the serrated pathway. These molecular features appeared to support the algorithm for CAs and SSAs. However, it is important to note as in the literature that this algorithm should be further validated with larger case series with molecular analysis.

The present study has some limitations. Firstly, the number of patients is low, and further studies with a larger sample size are needed to confirm the results. Secondly, molecular studies could not be performed on all cases due to the limited availability of tumoral and non-tumoral tissue blocks required for molecular analysis.

## CONCLUSION

In conclusion, our study examined the histopathological parameters required for diagnosis in various lesions and found that none of the criteria were specific to a single lesion. Molecular analysis also revealed unexpected mutations in the diagnostic groups, further supporting this finding. Cases with dysplasia and serrated features presented a diagnostic challenge. We observed that a threshold value of 25% for the serration parameter was significant, while other unexpected histopathological parameters were typically below this threshold. In a small number of cases, ectopic crypts and eosinophilic nuclei, features defined for TSAs, exceeded the threshold of 10%. Based on these findings, an algorithm can be developed. Using a threshold value of 25% for luminal serration can aid in differentiating between CAs and serrated lesions, while a threshold value of 10% for ectopic crypts and cylinder nuclei can serve as an auxiliary method to differentiate TSAs. The molecular data also appeared to support these findings.

## Conflict of Interest

The authors report there are no competing interests to declare.

## Funding

The study was carried out as a scientific research project (Project no: 2018/043) accepted by the Health Sciences University Scientific Research Projects Unit.

## References

[ref-1] Amin Mahul B., Greene Frederick L., Edge Stephen B., Compton Carolyn C., Gershenwald Jeffrey E., Brookland Robert K., Meyer Laura, Gress Donna M., Byrd David R., Winchester David P. (2017). The Eighth Edition AJCC Cancer Staging Manual: Continuing to build a bridge from a population-based to a more "personalized" approach to cancer staging. CA Cancer J Clin.

[ref-2] Nagtegaal Iris D., Odze Robert D., Klimstra David, Paradis Valerie, Rugge Massimo, Schirmacher Peter, Washington Kay M., Carneiro Fatima, Cree Ian A., WHO Classification of Tumours Editorial Board (2020). The 2019 WHO classification of tumours of the digestive system. Histopathology.

[ref-3] Andrea Mille Kyhn, Jepsen Rikke Karlin, Klein Mads Falk, Gögenur Ismail, Kuhlmann Tine Plato (2024). Predictors for dMMR colorectal cancer in patients with serrated lesions and polyps - A register-based cohort study. Cancer Epidemiol.

[ref-4] Odze Robert D., Goldblum John R. (2023). Surgical pathology of the GI tract, liver, biliary tract, and pancreas.

[ref-5] Jass J. R., Baker K., Zlobec I., Higuchi T., Barker M., Buchanan D., Young J. (2006). Advanced colorectal polyps with the molecular and morphological features of serrated polyps and adenomas: concept of a 'fusion' pathway to colorectal cancer. Histopathology.

[ref-6] Glatz Katharina, Pritt Bobbi, Glatz Dieter, Hartmann Arndt, O'Brien Michael J., Blaszyk Hagen (2007). A multinational, internet-based assessment of observer variability in the diagnosis of serrated colorectal polyps. Am J Clin Pathol.

[ref-7] Pai Rish K., Bettington Mark, Srivastava Amitabh, Rosty Christophe (2019). An update on the morphology and molecular pathology of serrated colorectal polyps and associated carcinomas. Mod Pathol.

[ref-8] Cenaj Odise, Gibson Joanna, Odze Robert D. (2018). Clinicopathologic and outcome study of sessile serrated adenomas/polyps with serrated versus intestinal dysplasia. Mod Pathol.

[ref-9] Torlakovic Emina, Skovlund Eva, Snover Dale C., Torlakovic Goran, Nesland Jahn M. (2003). Morphologic reappraisal of serrated colorectal polyps. Am J Surg Pathol.

[ref-10] Torlakovic Emina Emilia, Gomez Jose D., Driman David K., Parfitt Jeremy R., Wang Chang, Benerjee Tama, Snover Dale C. (2008). Sessile serrated adenoma (SSA) vs. traditional serrated adenoma (TSA). Am J Surg Pathol.

[ref-11] Al-Sohaily Sam, Biankin Andrew, Leong Rupert, Kohonen-Corish Maija, Warusavitarne Janindra (2012). Molecular pathways in colorectal cancer. J Gastroenterol Hepatol.

[ref-12] Snover Dale C. (2011). Update on the serrated pathway to colorectal carcinoma. Hum Pathol.

[ref-13] O'Brien Michael J., Zhao Qing, Yang Shi (2015). Colorectal serrated pathway cancers and precursors. Histopathology.

[ref-14] Fearon E. R., Vogelstein B. (1990). A genetic model for colorectal tumorigenesis. Cell.

[ref-15] Leggett Barbara, Whitehall Vicki (2010). Role of the serrated pathway in colorectal cancer pathogenesis. Gastroenterology.

[ref-16] Cappello Filippo, Angerilli Valentina, Dal Santo Luca, Munari Giada, Sabbadin Marianna, Lo Mele Marcello, Pennelli Gianmaria, Luchini Claudio, Parente Paola, Lazzi Stefano, Fassan Matteo (2022). Morphological and molecular characterization of colorectal sessile serrated lesions with dysplasia. Pathol Res Pract.

[ref-17] Bell Phoenix D., Anderson Joseph C., Srivastava Amitabh (2022). The Frontiers of Serrated Polyps. Am J Surg Pathol.

[ref-18] Chino Akiko, Kawachi Hiroshi, Takamatsu Manabu, Hatamori Hiroyuki, Ide Daisuke, Saito Shoichi, Igarashi Masahiro, Fujisaki Junko, Nagayama Satoshi (2020). Macroscopic and microscopic morphology and molecular profiling to distinguish heterogeneous traditional serrated adenomas of the colorectum. Dig Endosc.

[ref-19] Bateman Adrian C. (2021). The spectrum of serrated colorectal lesions-new entities and unanswered questions. Histopathology.

[ref-20] Ensari Arzu, Bilezikçi Banu, Carneiro Fatima, Doğusoy Gülen Bülbül, Driessen Ann, Dursun Ayşe, Flejou Jean-François, Geboes Karel, Hertogh Gert, Jouret-Mourin Anne, Langner Cord, Nagtegaal Irıs D., Offerhaus Johan, Orlowska Janina, Ristimäki Ari, Sanz-Ortega Julian, Savaş Berna, Sotiropoulou Maria, Villanacci Vincenzo, Kurşun Nazmiye, Bosman Fred (2012). Serrated polyps of the colon: how reproducible is their classification?. Virchows Arch.

[ref-21] Bettington Mark, Walker Neal, Rosty Christophe, Brown Ian, Clouston Andrew, McKeone Diane, Pearson Sally-Ann, Klein Kerenaftali, Leggett Barbara, Whitehall Vicki (2016). Serrated tubulovillous adenoma of the large intestine. Histopathology.

[ref-22] Cansiz Ersöz C., Kiremitci S., Savas B., Ensari A. (2020). Differential diagnosis of traditional serrated adenomas and tubulovillous adenomas: a compartmental morphologic and immunohistochemical analysis. Acta Gastroenterol Belg.

[ref-23] Chetty Runjan, Hafezi-Bakhtiari Sara, Serra Stefano, Colling Richard, Wang Lai Mun (2015). Traditional serrated adenomas (TSAs) admixed with other serrated (so-called precursor) polyps and conventional adenomas: a frequent occurrence. J Clin Pathol.

[ref-24] Kim Mi-Jung, Lee Eun-Jung, Suh Jung-Pil, Chun Sung-Min, Jang Se-Jin, Kim Do Sun, Lee Doo Han, Lee Suk Hee, Youk Eui Gon (2013). Traditional serrated adenoma of the colorectum: clinicopathologic implications and endoscopic findings of the precursor lesions. Am J Clin Pathol.

[ref-25] Hafezi-Bakhtiari Sara, Wang Lai Mun, Colling Richard, Serra Stefano, Chetty Runjan (2015). Histological overlap between colorectal villous/tubulovillous and traditional serrated adenomas. Histopathology.

[ref-26] Väyrynen Sara A., Väyrynen Juha P., Klintrup Kai, Mäkelä Jyrki, Tuomisto Anne, Mäkinen Markus J. (2016). Ectopic crypt foci in conventional and serrated colorectal polyps. J Clin Pathol.

[ref-27] Bettington Mark L., Walker Neal I., Rosty Christophe, Brown Ian S., Clouston Andrew D., McKeone Diane M., Pearson Sally-Ann, Klein Kerenaftali, Leggett Barbara A., Whitehall Vicki L. J. (2015). A clinicopathological and molecular analysis of 200 traditional serrated adenomas. Mod Pathol.

[ref-28] Hiromoto Takafumi, Murakami Takashi, Akazawa Yoichi, Sasahara Noriko, Saito Tsuyoshi, Sakamoto Naoto, Mitomi Hiroyuki, Nagahara Akihito, Yao Takashi (2018). Immunohistochemical and genetic characteristics of a colorectal mucin-rich variant of traditional serrated adenoma. Histopathology.

